# The interleukin-6 and noradrenaline mediated inflammation-stress feedback mechanism is dysregulated in metabolic syndrome: Effect of exercise

**DOI:** 10.1186/1475-2840-10-42

**Published:** 2011-05-20

**Authors:** Leticia Martín-Cordero, Juan J García, Maria D Hinchado, Eduardo Ortega

**Affiliations:** 1 Grupo de Investigación de Inmunofisiología, Departamento de Fisiología, Facultad de Ciencias, Universidad de Extremadura, Badajoz, Spain

## Abstract

**Background:**

Metabolic syndrome (MS) is a metabolic disorder associated with obesity, type-II diabetes, and "low grade inflammation", with the concomitant increased risk of cardiovascular events. Removal of the inflammatory mediator signals is a promising strategy to protect against insulin resistance, obesity, and other problems associated with MS such as cardiovascular disease. The aim of the present investigation was to determine the "inflammatory and stress status" in an experimental model of MS, and to evaluate the effect of a program of habitual exercise and the resulting training-induced adaptation to the effects of a single bout of acute exercise.

**Methods:**

Obese Zucker rats (fa/fa) were used as the experimental model of MS, and lean Zucker rats (Fa/fa) were used for reference values. The habitual exercise (performed by the obese rats) consisted of treadmill running: 5 days/week for 14 weeks, at 35 cm/s for 35 min in the last month. The acute exercise consisted of a single session of 25-35 min at 35 cm/s. Circulating concentrations of IL-6 (a cytokine that regulates the inflammatory and metabolic responses), CRP (a systemic inflammatory marker), and corticosterone (CTC) (the main glucocorticoid in rats) were determined by ELISA, and that of noradrenaline (NA) was determined by HPLC. Glucose was determined by standard methods.

**Results:**

The genetically obese animals showed higher circulating levels of glucose, IL-6, PCR, and NA compared with the control lean animals. The habitual exercise program increased the concentration of IL-6, PCR, NA, and glucose, but decreased that of CTC. Acute exercise increased IL-6, CRP, and NA in the sedentary obese animals, but not in the trained obese animals. CTC was increased after the acute exercise in the trained animals only.

**Conclusion:**

Animals with MS present a dysregulation in the feedback mechanism between IL-6 and NA which can contribute to the systemic low-grade inflammation and/or hyperglycaemia of MS. An inappropriate exercise intensity can worsen this dysregulation, contributing to the metabolic, inflammatory, and stress disorders associated with MS. Habitual exercise (i.e., training) induces a positive adaptation in the response to acute exercise.

## Background

Metabolic syndrome (MS) is a metabolic disorder associated with obesity, and involves risk factors for type-II diabetes mellitus and arteriosclerosis, with the concomitant increased risk of cardiovascular events [1-3]. Obesity is associated with "low-grade inflammation" [4-6], a term that is used to reflect increments in the systemic concentration of tumour necrosis factor-alpha (TNF-α), interleukin (IL)-1β, IL-6, IL-1ra, and C-reactive protein (CRP) [7]. IL-6, CRP, and TNF-α are the main inflammatory molecules associated with obesity-related inflammation. They are involved in the pathobiology of MS-associated disorders, such as obesity, insulin resistance, coronary heart disease, type-II diabetes, and hypertension [4,6,8-10]. Wellen and Hotamisligil (2005) proposed a model of interaction between the metabolic and immune systems. In this model, the relationship between obesity and inflammation appears to be two-way and involves positive feedback, i.e., inflammation *per se *induces obesity and insulin resistance, and in turn obesity induces low-grade inflammation and promotes insulin resistance and all of the other characteristics associated with MS [6]. Inflammatory cytokines can also stimulate the hypothalamus-pituitary-adrenal (HPA) axis, with, as a consequence, increased levels of glucocorticoids affecting inflammatory and immune processes. Disruption of the cytokine-HPA axis feedback loop can aggravate inflammatory pathologies [11]. It is also well established that noradrenaline (NA) is involved in regulating both metabolism and most of the mechanisms of the immune response, including the innate response and the systemic and local release of inflammatory cytokines [12-16,11] such as IL-6 [17,18]. Indeed, noradrenergic dysfunction, including over-activity of the sympathetic nervous system (SNS), is today also recognized as a characteristic of obesity-related MS, contributing to the pathophysiology and clinical prognosis [19,20].

Removal of the inflammatory mediator signals would seem to be a promising strategy to protect against insulin resistance, obesity, and the other problems associated with MS, such as cardiovascular diseases [21-23,6]. Exercise (with or without caloric restriction) has frequently been recommended for obese persons [24]. Indeed, habitual aerobic exercise is an accepted therapeutic strategy in the management of MS since it improves the diabetic status and insulin sensitivity, thus reducing the risk of cardiovascular disease [25]. Based on its anti-inflammatory effects [26], exercise can also be used as a means to control low-grade systemic inflammation [27]. Nevertheless, it is also clearly accepted that exercise is a form of stress, and that the relation between exercise, stress, and inflammation is a good model of neuroendocrine interaction. Exercise stimulates the innate immune responses, with its effects on the inflammatory response in particular being mediated by activation of the SNS and/or the HPA axis [28-30]. A well-controlled and regulated stimulation of the innate and/or inflammatory immune mechanisms during exercise can help prevent infection, but over-stimulation of the inflammatory response could also be harmful for people with inflammatory diseases [12,31,28,33], as might well be the case in MS and cardiovascular-associated disorders.

The obese Zucker rat (*fa/fa*) is the most commonly used animal model for the study of human type-II diabetes [34,35,10]. Given this context, the aim of the present investigation was to determine the "inflammatory and stress status" of obese Zucker rats compared to healthy lean rats used as control, and to evaluate the effect of a program of habitual exercise performed by the obese rats in the form of training-induced adaptation to the effects of a single session of acute exercise.

## Methods

### Animals and experimental design

45 male Zucker rats (Harlan, United Kingdom) were used, comprising 36 obese rats (HsdOla:ZUCKER-*Lepr^fa^*, homozygous *fa/fa*) as the experimental model for the MS study, and 9 lean rats (heterozygous *Fa/fa*) as the healthy controls for reference values. Obese Zucker rats carry a spontaneous obesity-causing mutation in the leptin receptor gene (*fa*) (*Lepr^fa ^*is an autosomal recessive mutation on chromosome 5). They exhibit obesity at 4 to 5 weeks of age (680 ± 5 g obese vs 421 ± 8 g lean rats immediately before sacrifice at 6 months of age), and share many metabolic characteristics with human obesity-associated type-II diabetes such as insulin-resistance, hyperphagia, hyperlipidaemia (developing adipocyte hypertrophy and hyperplasia), hypercholesterolaemia, muscle atrophy, and hyperinsulinaemia (as specified by Harlam). At the beginning of the experiment, the rats were 2 months old, and were housed in cages with food (A04 diet, SAFE, France) and water *ad libitum *in the animal facilities of the Medical Faculty of the Autonomous University of Madrid. The animals were undisturbed until they were 10-12 weeks old. Then, the experimental design was targeted at studying the differences between the lean and obese rats, the effects of habitual exercise or training on the obese rats, and the responses to a single bout of acute exercise performed by the obese sedentary or obese trained animals.

The obese Zucker rats (n = 36) were divided into 4 experimental groups: sedentary rats (n = 9), sedentary rats that performed a single bout of acute exercise (n = 9), trained rats (n = 9), and trained rats that performed a single bout of acute exercise (n = 9). All animals were killed at approximately 6 months of age. Given that rats are animals with nocturnal activity, the light/dark cycle was adapted for the animals to perform exercise during their active phase, with the onset of the dark phase about 2 hours before the start of the training sessions (light 01:30 - 13:30 h; dark 13:30-01:30 h). The animals were trained habitually between 15:00 and 18:00 h. The trained animals were kept at rest for 72 hours prior to collection of blood or to the performance of the bout of acute exercise. The blood samples following these bouts of acute exercise were collected immediately at the conclusion of the session. Sedentary lean Zucker rats also 6 months old (n = 9) were used as controls to get reference values in healthy animals. At 08:30 on the day the animals were killed, feed was withdrawn from all the animals.

Blood samples were collected from anaesthetized animals. The circulating concentrations of IL-6, CRP, NA, and corticosterone (CTC) were determined in each animal. The comparison of the results between the obese and the lean Zucker rats revealed the differences in the inflammatory and stress biomarkers evaluated in this experimental model of MS, and the comparison between the different experimental groups of obese animals revealed the immunophysiological adaptations and responses to physical exercise.

### Exercise

The habitual exercise training consisted of running on a treadmill (Li 870, Letica Scientific Instruments, Barcelona, Spain) with no slope, based on adaptation, progression, and maintenance phases in intensity and duration from 25 cm/s for 10 min in the first weeks to values close to 35 cm/s for 35 min in the last month. The exercise was performed 5 days per week, for 14 weeks. The bout of acute exercise consisted of running on the treadmill for 5 min at 17 cm/s followed by 25-35 min at 35 cm/s, with no slope.

### Ethical approval

The experiment was approved by the Ethical Committees of the Autonomous University (Madrid, Spain) and of the University of Extremadura (Badajoz, Spain) according to the "Principles of Laboratory Animal Care" (NIH publication No. 86-23, revised 1985) and, the guidelines of the European Community Council Directives and the Declaration of Helsinki.

### Anaesthesia and collection of serum and plasma

The fasted animals were gas anaesthetized with isoflurane (AErrane, Baxter, Valencia, Spain), with a starting dose of 2% oxygen and 3.5% isoflurane, and maintenance dose of 1.5% oxygen and 3% isoflurane. Blood was extracted from the live animals, drawn from the abdominal mesenteric vein using heparinized syringes. One additional drop was taken for the direct estimation of glycaemia using Accutrend strips (Roche). For the serum isolation, the blood was deposited in tubes without anticoagulant at room temperature for 30 min and then centrifuged at 700 g for 10 min at room temperature. For the plasma isolation, the blood was deposited in tubes with 0.1 volumes of cold anticoagulant (26 mM of EGTA and 130 mM DTT) and centrifuged at 900 g for 7 min.

### Plasma noradrenaline determination

Plasma samples from each animal were stored at -80°C until assay. Noradrenaline concentration was determined by HPLC (Electrochemical Detection, Coulochem) using a commercial kit (Chromosystems Instruments and Chemical, GMBH, Munich, Germany). During the extraction of the samples, an internal standard (dihydroxybenzylamine) was added to allow the subsequent calculation of the exact original concentration avoiding losses during the process. The column used was a C18 (Waters), the working potential was between 450 and 660 mV, the flow was 1 ml/min, and the pressure did not exceed 200 bar. After injection of 20 μl of the final processed sample, a chromatogram was obtained in which one peak was observed at a lag of approximately 4 min, corresponding to noradrenaline.

### Serum CRP, IL-6, and CTC determinations

Serum samples from each animal were stored at - 80°C until assay. The CTC and CRP concentrations were determined by a double polyclonal antibody sandwich enzyme immunoassay (EIA) specific for rats (DRG Instruments GmbH, Germany for CTC; Chemicon International, USA for CRP), and IL-6 concentrations by a solid-phase sandwich enzyme-linked immunosorbent assay (ELISA) specific for rats (Biosource Immunoassay, Nivelles, Belgium). Samples were assayed at optimal concentrations and according to the manufacturer's instructions.

### Statistical analysis

Values are given as mean ± SEM. The variables were normally distributed (tested by the normality test). Student's *t*-test was used for comparisons between the pairs of groups (non-paired samples). A non-parametric ANOVA and Scheffe's *F*-test was used for comparisons among the three experimental groups. The minimum significance level was taken to be *p*<0.05.

## Results

**Figure 1:**
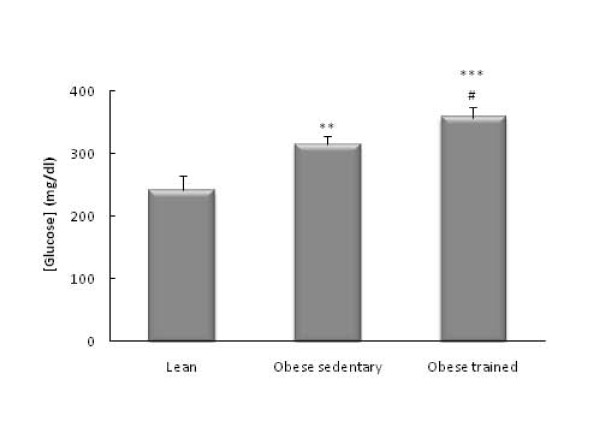
**Circulating levels of glucose in obese Zucker rats: effect of habitual exercise (control lean rats are used as reference values)**. Each column represents the mean ± SEM of the values obtained in 9 animals of each experimental group. ** p < 0.01, *** p < 0.001 lean *vs *obese rats; # p < 0.05 sedentary *vs *trained obese rats.

**Figure 2:**
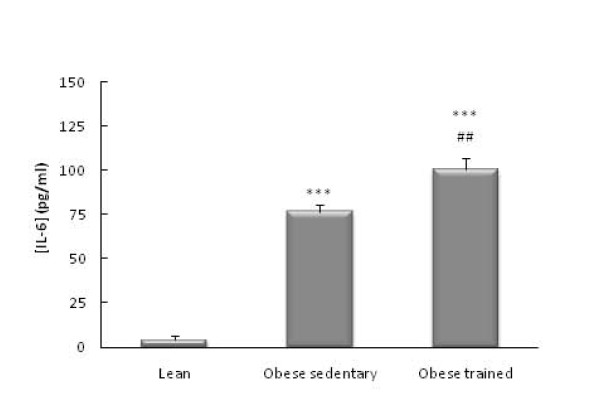
**Circulating levels of IL-6 in obese Zucker rats: effect of habitual exercise (control lean rats are used as reference values)**. Each column represents the mean ± SEM of the values obtained in 9 animals of each experimental group. *** p < 0.001 lean *vs *obese rats; ## p < 0.01 sedentary *vs *trained obese rats.

**Figure 3:**
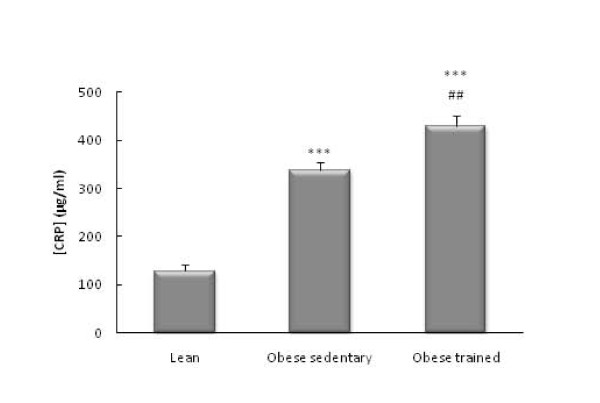
**Circulating levels of CRP in obese Zucker rats: effect of habitual exercise (control lean rats are used as reference values)**. Each column represents the mean ± SEM of the values obtained in 9 animals of each experimental group. *** p < 0.001 lean *vs *obese rats; ## p < 0.01 sedentary *vs *trained obese rats.

**Figure 4:**
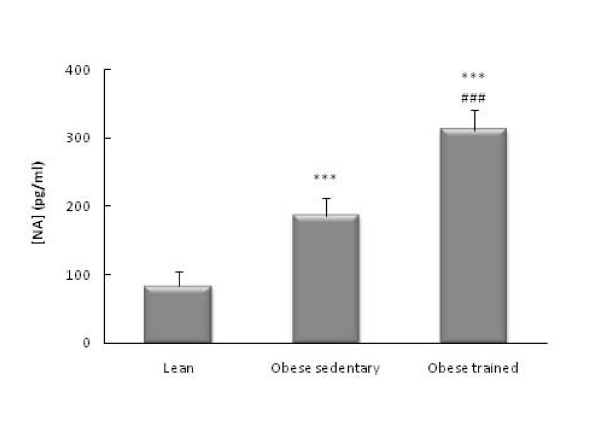
**Circulating levels of NA in obese Zucker rats: effect of habitual exercise (control lean rats are used as reference values)**. Each column represents the mean ± SEM of the values obtained in 9 animals of each experimental group. *** p < 0.001 lean *vs *obese rats; ###p < 0.001 sedentary *vs *trained obese rats.

**Figure 5:**
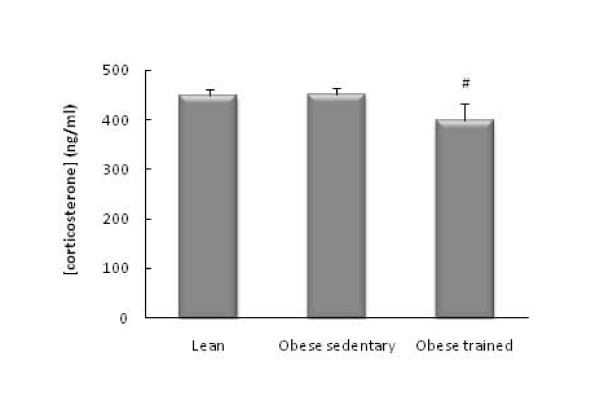
**Circulating levels of corticosterone in obese Zucker rats: effect of habitual exercise (control lean rats are used as reference values)**. Each column represents the mean ± SEM of the values obtained in 9 animals of each experimental group. #p < 0.05 sedentary *vs *trained obese rats.

The obese Zucker rats had higher circulating levels of glucose (*p*<0.01, Figure 1), IL-6 (*p*<0.001 Figure 2), CRP (*p*<0.001, Figure 3), and NA (*p*<0.001, Figure 4) than the lean rats. There were no significant differences in the circulating concentrations of CTC (Figure 5) probably because of the high levels presented by the Zucker strain in comparison with other rat strains (such as Wistar, which in our laboratory present values of around 50 ng/ml). One also observes in these figures that the obese animals which performed the habitual exercise protocol for 14 weeks had higher circulating levels of glucose (*p*<0.05, Figure 1), IL-6 (*p*<0.01 Figure 2), CRP (*p*<0.01, Figure 3), and NA (*p*<0.001, Figure 4) than the sedentary obese animals, but lower levels of CTC (*p*<0.05, Figure 5).

Table 1 presents the effects of a bout of exercise on the sedentary and trained obese rats. One observes that the effect on the sedentary obese animals was to increase their circulating levels of IL-6 (*p*<0.001), CRP (*p*<0.05), and NA (*p*<0.01) relative to those which did not perform this bout of exercise. But no such stimulation of the three markers was observed in the trained obese animals. Indeed, these animals showed a slight (although not significant) decrease in the concentration of these markers in response to the bout of exercise. This difference between the trained and sedentary animals was especially notable in the case of IL-6, with a decline to a value that was significantly lower (*p*<0.05) than that of the sedentary animals which performed the bout of acute exercise. This occurred in parallel with an increase in the circulating concentration of CTC. There were no significant changes in glucose levels in response to the bout of exercise in either group of obese animals.

**Table 1:** Effect of acute exercise on metabolic, inflammatory, and stress biomarkers in the obese Zucker rats: Training-induced adaptation.

	Obese sedentary rats		Obese trained rats	
	
	without acute exercise	with acute exercise	without acute exercise	with acute exercise
Glucose (mg/dl)	314.4 ± 12	289.5 ± 15	357.5 ± 15	364.6 ± 17
NA (pg/ml)	186 ± 26	319 ± 47 **	311 ± 29	283 ± 42
IL-6 (pg/ml)	76.5 ± 4.2	128 ± 14.7 ***	100 ± 6.2	88 ± 9.9 **#**
CRP (μg/ml)	338 ± 17	390 ± 9 *	430 ± 22	412 ± 19
Corticosterone	451 ± 13	468 ± 11	408 ± 33	478 ± 6●

Each number is the mean ± SEM of the values obtained in 9 animals of each experimental group. *p < 0.05, **p < 0.01, ***p < 0.001 obese sedentary rats without acute exercise *vs *obese sedentary rats after acute exercise; #p < 0.05 *vs *obese sedentary rats after acute exercise; ●p < 0.05 *vs *obese trained rats without acute exercise.

## Discussion

### Dysregulation in stress and inflammatory biomarkers in MS

It is currently accepted that neuro-immuno-endocrine disorders can play a role in obesity, hypertension, and insulin resistance, and that neuro-immuno-endocrine dysfunction is associated with abnormalities in the inflammatory response or with the local over-activity of pro-inflammatory factors [13]. The high systemic levels of IL-6 and CRP in the obese animals of the present study, together with the high concentration of IL-1β also found in this animal model [35], confirm the low-grade inflammation that occurs in MS [4-6,36]. Similar results for CRP and IL-6 have been reported in the literature, also in Zucker diabetic rats [8,9]. In addition to their "inflammatory status", our obese animals presented a state of hyperglycaemia and systemic stress manifest in their higher concentrations of glucose and NA compared with the lean rats used as controls. We found no differences, however, in CTC concentrations, probably because of the Zucker strain's surprisingly high concentration of this hormone (and of IL-6), which could indicate that this strain has a dysregulated IL-6-CTC loop.

It has been reported that NA affects IL-6 release, in both inhibition and activation [17,18]. Although a different behaviour in pathological conditions such as MS cannot be ruled out, in healthy individuals NA generally causes decreases/increases in the systemic concentrations of inflammatory/anti-inflammatory cytokines, respectively [12,37]. Also IL-6, together with IL-1β, can stimulate the HPA axis and the SNS, and therefore the release of NA and glucocorticoids [38,11,12]. The stress system activated by the immune system thus stimulates a negative feedback mechanism that protects the organism from an excess of inflammatory proteins. Nonetheless, under conditions of stress and in pathological inflammatory conditions, NA may induce a rise in the systemic levels of IL-6 [39]. The high levels of NA and IL-6 (and IL-1β [35]) found in obese animals may reflect defective regulation of the negative inflammatory/stress feedback loop in MS - a physiological state that may in turn be either the cause or the consequence of the diabetes associated with obesity. Although the CTC levels determined in this study do not allow us to clearly conclude that the inflammatory cytokine-HPA axis feedback is dysregulated in obesity, the results do suggest reduced responsiveness to high levels of IL-6 in corticosterone release in the obese Zucker rats.

The multifunctional cytokine IL-6 is clearly involved in the regulation of metabolism, with confirmation of the link between obesity and inflammation [40-42]. Although IL-6 has traditionally been reported to play an important role in the pathogenesis of coronary artery disease and atherosclesoris, the possibility that IL-6 has many beneficial effects on health has been put forward recently. In this sense, IL-6 should not be considered good or bad, but as a molecule with both beneficial (particularly during exercise as discussed below) and destructive effects [43]. The release of IL-6 into the systemic circulation, and the fact that this release was observed to be greater in the obese Zucker rats, lends support to a recent suggestion that IL-6 plays a role as a systemic regulator of body weight and lipid metabolism [42]. There is still considerable controversy, however, about the effects of raised systemic levels of IL-6. A few years ago, this situation was linked to the development of diabetes, hypertension, and hyperlipidaemia [40]. Thus, it has been suggested that circulating IL-6 plays an important role in the development of insulin resistance [44] and atherosclerosis through its effects on metabolism: reducing hepatic insulin sensitivity and glucose uptake by adipocytes, and causing raised plasma insulin levels, hyperglycaemia, and hyperlipidaemia (reviewed by Eder and co-workers [42]). Other studies argue for IL-6 having a lipolytic role [45-47,27], with its participation in lipolysis and fat oxidation [46,48]. The controversy was sharpened with the observation that IL-6 knock-out mice develop mature-onset obesity, with hypertriglyceridaemia, glucose intolerance, and other features of MS [45] and the contrary results of a subsequent study [49] which observed no such phenomenon, probably because the comparison was made with different control strains [42]. It is now known that the cellular response to IL-6 depends on the metabolic state of the cell as well as on a combination of other external stimuli [50,51]. Therefore, to help resolve this controversy concerning the inflammatory (pro- or anti-inflammatory) and endocrine (whether or not inducive of insulin resistance, and therefore hypo-or hyper-glycaemic) effects of IL-6 in MS, it is vital to clarify whether inflammation is the cause or the consequence of metabolic dysregulation. In view of the overall results of the present study on the systemic levels of stress and inflammation markers, in our opinion it would be most plausible to hypothesize that elevated circulating levels of IL-6 and NA are the origin (and a good marker) of metabolic and "stress/neuroendocrine" inflammatory dysregulation in MS. The elevation of IL-6 would induce increased systemic release of NA (and probably of CTC also), which in turn could stimulate (or at least not inhibit) the release of IL-6 (or *vice versa*), and both NA and IL-6 might stimulate the release of glucose into the bloodstream. Elevated levels of IL-6 would also induce an increased hepatic release of CRP, a manifestation of pro-inflammatory status in MS. Therefore, this experimental model of MS presents, in addition to a hyperglycaemic state, a dysregulated inflammatory, neuroendocrine, and stress feedback system. Such dysregulation has already been indicated as present in other diseases of an inflammatory nature, with involvement of the inflammatory cytokines, catecholamines, and glucocorticoids released through the activation of the SNS and the HPA axis [12,39,11].

### Effect of physical exercise on stress/inflammation dysregulation in metabolic syndrome (MS)

Exercise is a form of physiological stress, and the relationship between it, stress, and inflammation is a good model of neuroendocrine interaction. The effects of exercise on the inflammatory response are primarily mediated through the SNS and/or the HPA axis [52,28-31]. Currently, regular physical activity is advocated as an effective non-pharmacological form of intervention in the treatment of several of the major disorders of MS. It has been shown to improve dyslipidaemia, insulin sensitivity, and the immune response to antigens [53-56,10]. It is therefore important to determine whether many of the benefits for MS attributed to exercise may be partly explained by its effect on mediators of the inflammatory system and of stress, such as the biomarkers studied in the present work. For example, in obese Zucker rats, the beneficial effect of exercise training on obesity and diabetes has been linked to anti-inflammatory mechanisms, with improvements in the circulating levels of CRP, adiponectin [8], IL-6, and TNF-α [9]. This suggests that regular exercise reduces systemic inflammation in MS, and that these inflammatory benefits are also correlated with improvements in several of the characteristics associated with MS. The present results showed, however, that the obese rats performing regular exercise have even higher circulating levels of NA, IL-6, and CRP than the sedentary obese rats.

During situations of exercise-induced stress (of particular importance in individuals with inflammatory pathologies), NA stimulates the systemic IL-6 concentration unrelated to inflammation [39]. Thus, the exercise-induced increase in the systemic concentration of IL-6 in the trained obese rats is probably noradrenaline mediated. These rats' elevated circulating levels of IL-6 could in turn stimulate a greater release of NA through the activation of the SNS, and the two molecules together (in a dysregulated interaction in MS) would contribute to the observed higher glucose levels. Another contribution to the higher concentration of IL-6 in the trained obese rats could also come from their lower concentration of CTC than in the sedentary obese animals, partially reducing the inhibitory effect of glucocorticoids on the release of IL-6. Furthermore, the elevated levels of IL-6 may also be contributing to increasing the systemic levels of the inflammatory marker CRP in the trained obese animals, since IL-6 is one of the most potent inducers of CRP release from the liver [7] and it can also induce the release of CRP from adipose tissue in MS [57]. The high concentration of CRP in the trained animals of the present study suggests that the exercise evaluated may in itself have a pro-inflammatory rather than an anti-inflammatory effect. The high glucose levels observed in the animals at the end of the habitual exercise protocol may be associated with the high levels of IL-6 and CRP. These, together with the high levels of NA, could explain the lack of improvement in insulin-resistance presented by these MS animals, and may even contribute to exacerbating this pathology and hence increase cardiovascular risks. One must be cautious about using exercise in "inflammatory pathologies": if the intensity of the exercise were inappropriate, instead of anti-inflammatory effects, it might induce pro-inflammatory effects, with potentially serious consequences in pathologies with an underlying dysregulation of the inflammatory mechanisms, such as is the case of MS as has been demonstrated by the results of the present study.

The high levels of NA might be stimulating the innate and/or inflammatory immune system, since it has been postulated that the NA released during physical exercise may act as a "stress mediator" or "danger signal" in situations in which homoeostasis may be altered, stimulating the innate and/or inflammatory immune responses and thus being able to alert the organism to possible attack by pathogens [58,28,30]. This would be consistent with the improved anti-pathogen response found in these animals after regular exercise [10,35]. Besedovsky & Del Rey (2007), in the very interesting view they present of how the immune system functions, argue that, while immune responses are physiological responses expected to be maximally efficient during infectious/inflammatory diseases, they sometimes contribute to pathology, since immunoregulation is itself a physiological process that operates simultaneously and interwoven with pathological events. Thus, one can conclude from the present results that the training protocol used (suitable and accepted for healthy animals to improve their innate immune response against pathogens) leads to a situation of stress and inflammation in MS animals, manifest in their raised circulating levels of NA, and that these could contribute (or *vice versa*) to increasing the concentration of IL-6 and CRP that will ultimately affect the regulation of glucose homoeostasis in these obese animals.

Finally, prior regular exercise or training induced a physiological adaptation in the obese animals to their confrontation with a single bout of exercise, which instead exacerbated the pro-inflammatory (increased systemic concentration of CRP and IL-6) and stress (increased systemic levels of NA) status of the sedentary obese animals. Nevertheless, considering overall the results for the trained animals, the decrease in NA and increase in CTC concentrations would contribute to reducing the systemic concentration of IL-6, and therefore also of CRP. Indeed, these results agree with the idea that, during situations of exercise-induced stress, catecholamines can stimulate IL-6 release and glucocorticoids can inhibit it [33,39]. In addition, the results overall showed that, for the MS animals, the single bout of physical exercise only had an anti-inflammatory effect in those that had been previously trained. Again, the key could be IL-6, since its decline in levels may reduce the release of NA and CRP, and allow a reduction in the levels of CTC.

## Conclusions

The animals with MS presented a dysregulation in the negative feedback mechanism between IL-6 and NA (and probably also CTC) that can contribute to the systemic low-grade inflammation and/or hyperglycaemia of MS. An inappropriate intensity of exercise can worsen this dysregulation, increasing the levels of NA, IL-6, and CRP, and contributing to the metabolic, inflammatory, and stress disorders associated with MS. It appears to be important to determine whether the origin of this dysregulation is an increase in the release of IL-6 or NA so as to understand the effects of regular physical exercise.

Given its pleiotropic effects, regular physical activity or training has been proposed as a "polypill" capable of replacing, or at least reducing, the use of drugs to control the cardiovascular risks presented by obese type-II diabetes patients [59]. In considering this suggestive proposal, however, it needs to be emphasized that, as would plainly be accepted for any "pharmacological drug", it is very important to individualize the "dose" of the exercise treatment for each patient so as to avoid the possible side effects of exercise such as unhealthy pro-inflammatory and stress responses, since alterations in the inflammatory-stress feedback loop can themselves worsen MS.

Obviously, without ignoring the difficulty of extrapolating directly to humans, our present study in obese Zucker rats is a further warning for patients diagnosed with diabetes/MS who frequently start exercise programs that might not be appropriate, thus increasing the risk of deterioration of their condition due to increased dysregulation of stress/inflammation feedback mechanisms.

## List of abbreviations

CRP: C-reactive protein; CTC: corticosterone; IL: interleukin; NA: noradrenaline; MS: metabolic syndrome; HPA: hypothalamus-pituitary-adrenal; DM2: type-II diabetes mellitus; SNS: sympathetic nervous system

## Competing interests

The authors declare that they have no competing interests.

## Authors' contributions

All authors have read and approved the final version of the manuscript. LMC performed the experimental work of the paper. These results form part of her doctoral thesis (supervised by Dr Ortega). She also contributed to writing the manuscript and the discussion of the results. JJG collaborated actively in all the experimental work. MDH collaborated actively in all the experimental work. EO is Director of the Immunophysiology Research group to which all the authors belong. He designed the experiments, supervised the experimental work, prepared the discussion of the results, and wrote the main part of the manuscript. He is the principal investigator of the grants.
